# Expansion of Moso bamboo into Chinese fir stands persistently depletes rhizosphere bioavailable P pools: a seasonal, space-for-time approach

**DOI:** 10.3389/fpls.2025.1718574

**Published:** 2026-01-20

**Authors:** Shuangbo Bi, Xuerong Shi, Chunju Peng, Tianyi Hu, Jing Chen, Jingchen Xie, Haicheng Li, Tingting Cao, Man Shi, Zhikang Wang, Quan Li, Xinzhang Song

**Affiliations:** 1 State Key Laboratory for Development and Utilization of Forest Food Resources, Zhejiang A&F University, Hangzhou, China; 2 Wenzhou Key Laboratory of Early Sprouting Tea Breeding, Wenzhou Academy of Agricultural Sciences, Wenzhou, China

**Keywords:** forest succession, phosphorus fractions, rhizosphere soil, soil pH, subtropical forest

## Abstract

**Introduction:**

Accurate understanding of soil phosphorus (P) fractions is crucial for enhancing plant productivity and deciphering forest succession patterns; however, the dynamics of rhizosphere soil P fractions and their influencing factors during forest succession or land-type conversion, particularly in highly weathered tropical and subtropical regions, have not been comprehensively elucidated.

**Methods:**

Using a space-for-time replacement strategy, in this study, we examined how Moso bamboo (*Phyllostachys edulis*) expansion into Chinese fir (*Cunninghamia lanceolata*) forests affects P fractions in rhizosphere soil across various seasons within a subtropical region. The research focused on seasonal variations in soil P dynamics resulting from this invasive expansion. We further evaluated key drivers, encompassing soil physicochemical characteristics and microbial traits.

**Results and discussion:**

Compared to pure Chinese fir forests, mixed bamboo–fir stands had significant reductions in total P (excluding spring), CaCl_2_-P, Citrate-P, Enzyme-P (excluding spring), and HCl-P (excluding winter) throughout the seasonal cycle (*p* < 0.05). Pure bamboo forests showed further reduction in total P, Citrate-P, Enzyme-P, and HCl-P, along with reduced CaCl_2_-P (except summer and winter) (*p* < 0.05), with most P fractions (except CaCl_2_-P in summer, Citrate-P and HCl-P in summer and autumn, and Enzyme-P in summer) being lower in these stands than in mixed forests, which showed a decreasing trend with increasing expansion intensity. CaCl_2_-P, citrate-P, and HCl-P levels were consistently higher in summer and autumn than in winter and spring across Moso bamboo, Chinese fir, and mixed forest stands. Variations in P fractions were under the major control of nitrogen components and soil pH. This study highlights the importance of clarifying P fraction dynamics to understand forest succession mechanisms and informing P management strategies for enhancing forest productivity.

## Introduction

Phosphorus (P) is a critical limiting factor for ecosystem productivity and function, playing fundamental roles in key physiological processes, including anabolism, catabolism, energy transfer, and cellular homeostasis-that collectively underpin plant development and soil fertility (Bai et al., 2024; Jian et al., 2022; Paz-Ares et al., 2022; Yang et al., 2024; Ye et al., 2021). Plants acquire P mainly from the soil, whose P composition is highly complex (Bai et al., 2023; Zhang et al., 2025b). Therefore, accurate characterization of soil P fractions is crucial for improving plant P-use efficiency and understanding P cycling dynamics (Guan et al., 2024; Park et al., 2022). However, traditional approaches, such as the Hedley fractionation method, have emphasized chemical extraction but often overlooked plant acquisition pathways (DeLuca et al., 2015; Hedley and Stewart, 1982). Recently, the bioavailable P fractionation methodology has been crafted, allowing for the sorting of soil P according to how plants absorb it; this includes CaCl_2_-P, which plants readily take up, Citrate-P, which is inorganic P activated by organic acids secreted by roots, Enzyme-P, which is organic P broken down by phosphatase enzymes, and HCl-P, the pool of P that can be released through proton shedding (DeLuca et al., 2015). Applying this approach to different forest types offers a more ecologically relevant understanding of P speciation and insights into plant P-acquisition strategies.

Tropical and subtropical forests make up roughly 45% of the global woodlands and store approximately 72% of the carbon (C) in woodland biomass, underscoring their essential role in mitigating anthropogenic CO_2_ emissions and supporting C neutrality (FAO, 2020; Wang et al., 2024). In these regions, the warm and humid climate promotes the immobilization of soil P through fixation with iron and aluminum oxides, resulting in limited P availability for plants (Kochian, 2012). Moso bamboo (*Phyllostachys edulis*) and Chinese fir (*Cunninghamia lanceolata*) are important forest resources in the subtropical region of China (Li et al., 2024; Wu et al., 2019; Wang et al., 2025; Zhang et al., 2024, Zhang et al., 2025a). The area of Moso bamboo forests is approximately 5.28 million hectares, with an annual C sequestration capacity of approximately 6.1–7.3 t C·ha^-1^ (Li et al., 2025; Lv et al., 2025; Song et al., 2017, Song et al., 2020). Chinese fir plantations cover over 9.9 million hectares, with a total standing volume of 755 million cubic meters (Lv et al., 2024). Driven by both natural dispersal and weakened human management (Li et al., 2022; Xu et al., 2020), Moso bamboo, with its clonal growth characteristics (Kotangale et al., 2025; Zhang et al., 2024, 2025a), continues to expand into Chinese fir forests and other forest types (Bai et al., 2016b; Jiang et al., 2024). Therefore, elucidating the changes in soil P fractions during Moso bamboo expansion is crucial not only for understanding the evolution of P cycling in the context of forest succession but also for assessing and enhancing regional forest C sink functions.

The expansion of Moso bamboo significantly alters soil properties, including pH (Ouyang et al., 2022), enzyme activities (Liu et al., 2021), nitrogen (N) mineralization rates (Song et al., 2016a; Wu et al., 2019), C and N cycling (Bai et al., 2016a; Chen et al., 2021; Li et al., 2017), and microbial community structure and function (Liu et al., 2021). Such biological and environmental variable shifts are expected to profoundly influence soil P fractions. Furthermore, the expansion of Moso bamboo has been found to alter the contents of CaCl_2_-P, HCl-P, and Enzyme-P in bulk soil (Song et al., 2016b; Yang et al., 2024). However, these studies have largely overlooked the rhizosphere soil. Although rhizosphere P pools are generally smaller in size than those in bulk soil, they exhibit higher turnover rates (Qetrani et al., 2024). Consequently, even minor changes in these rhizosphere pools induced by plant expansion may significantly affect plant-available P supply. This highlights the need for in-depth research into rhizosphere P cycling processes in the context of plant expansion. Additionally, Moso bamboo, as a fast-growing clonal plant, possesses a dense, shallow rhizome-root system and an aggressive growth strategy with high nutrient demands (Peng et al., 2021; Wang et al., 2025). In contrast, Chinese fir is a deep-rooted tree species with relatively slow growth and a more conservative nutrient use strategy (Yan et al., 2019). This sharp contrast in growth rate, resource acquisition strategy, and root spatial distribution makes their rhizospheres a highly representative model system for investigating how plant expansion differentially drives soil P cycling processes and underlying microbial mechanisms. Studying this specific interface can provide key insights into the belowground interactions and resource competition between expanding and native tree species. Moreover, given that plants may employ seasonally dependent P-acquisition strategies, temporal variations in rhizospheric P dynamics have not been comprehensively elucidated. Therefore, investigating seasonal variations in rhizosphere soil P fractions amidst Moso bamboo’s expansion of Chinese fir forests is essential for elucidating the P-driven mechanisms underlying subtropical forest succession.

To address this gap, we chose a space-for-time substitution methodology (representing a chronosequence from Chinese fir forest to the bamboo expansion front and mature bamboo stands) to investigate the seasonal variations of soil P fractions and their driving factors. We hypothesized that (1) P fractions would diminish along the expansion gradient, driven by bamboo’s highly efficient P acquisition mechanisms (e.g., organic acid exudation and phosphatase activation), and (2) seasonal variations would dominate the spatiotemporal heterogeneity of P fractions by modulating soil properties and plant demand. Overall, the research offers a crucial understanding of how bamboo expansion affects biogeochemical processes, offering valuable knowledge for crafting strategies to minimize its ecological footprint and promote responsible forest stewardship.

## Materials and methods

### Study site and experimental design

**Figure 1:**
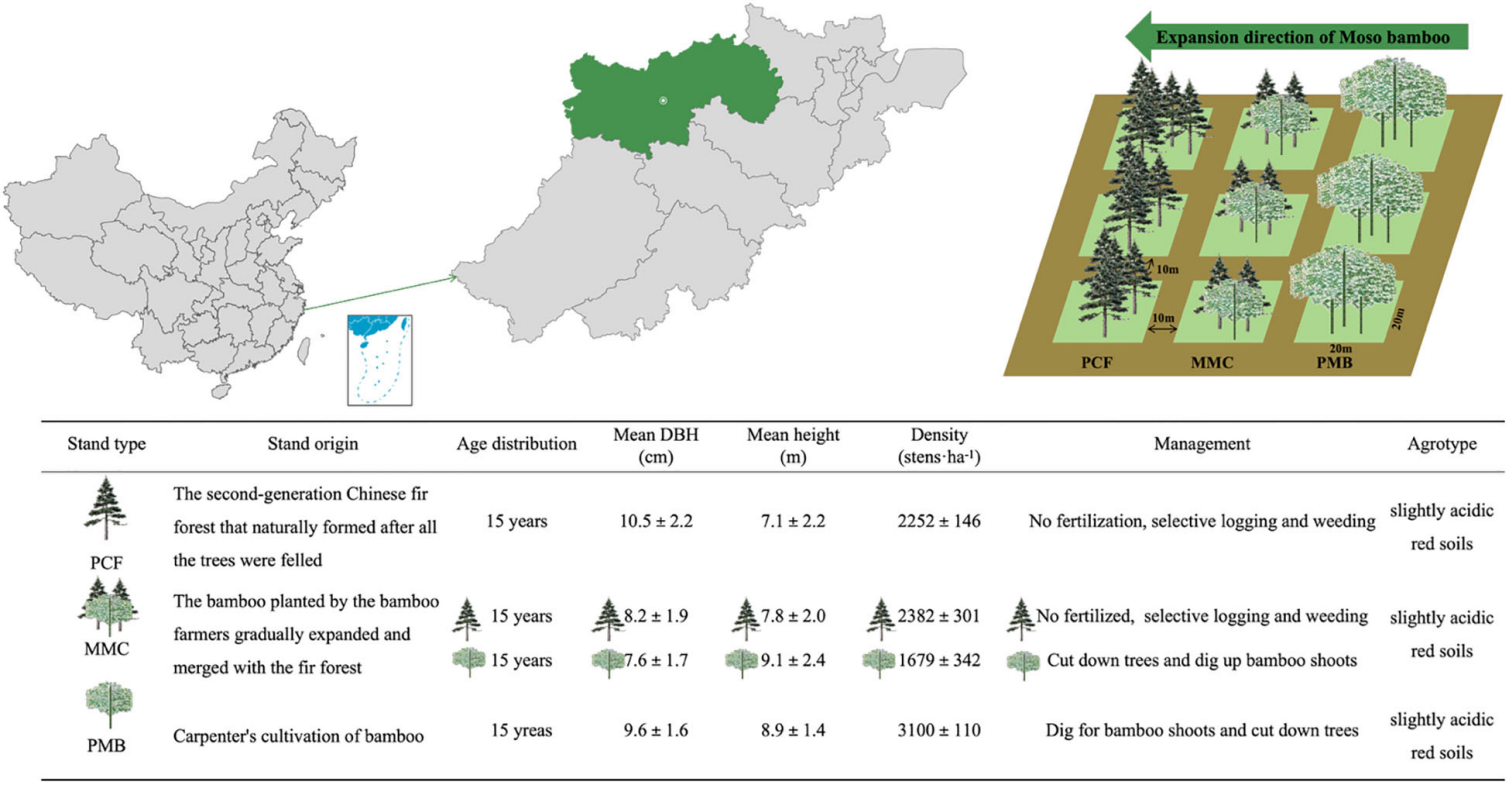
Schematic illustrating the experimental design: site locations and basic stand characteristics. DBH, diameter at breast height; PCF, the pure Chinese fir forest; MMC, the mixed Moso bamboo - Chinese fir forest; PMB, pure Moso bamboo forest.

The research was performed at Banqiao Town (30°10’N, 119°45’E), Lin’an District, China (**Figure 1**), situated in a subtropical monsoon weather zone, characterized by a mean annual temperature of 17.5 °C and annual precipitation ranging from 1350 to 1500 mm. Soils are slightly acidic red soils (Ultisols) derived from siltstone. Around 2004, pure Chinese fir stands replaced local evergreen broad-leaved forests. The subsequent Moso bamboo–Chinese fir mixed forest developed through bamboo expansion into fir stands. The selected pure Chinese fir forests (PCF) were even-aged stands approximately 15 years old, representing a mature stage prior to significant bamboo invasion. The Moso bamboo-Chinese fir mixed forests (MMC) represented an intermediate invasion stage, with bamboo having established for approximately 15 years. The pure Moso bamboo forests (PMB) represented a late invasion stage, where bamboo has been dominant for over 15 years (old bamboo naturally die or be cut down). This age sequence underpins our space-for-time substitution approach.

In June 2021, sample transects were established utilizing a space-for-time substitution approach, aligned with the directional extension of Moso bamboo plantations, adopting pure Chinese fir stands and pure Moso bamboo stands as reference boundaries. Three forest types were selected along each transect: (1) PCF, (2) MMC (fir-to-bamboo density ratio: 3:2), and (3) PMB. Three typical sample plots measuring 20 m × 20 m were developed for each forest species under comparable site conditions, resulting in nine sites. A 10-m-wide buffer zone was preserved between neighboring plots. **Figure 1** illustrates the configuration of the experimental plots and the features of the stand.

### Sampling collection

Samples of the root systems and rhizosphere soil were gathered from Moso bamboo and Chinese fir forest stands during summer (2021.07), autumn (2021.10), winter (2022.01), and spring (2022.04). Within each plot, three Chinese fir in PCF (Mean diameter at breast height (DBH)= 10.5cm) and MMC (Mean DBH = 8.2 cm) and Moso bamboo in MMC (Mean DBH = 7.6 cm) and PMB (Mean DBH = 9.6 cm) were selected based on comparable DBH (**Figure 1**). After removing surface litter, roots and rhizosphere soil were excavated from four cardinal directions per tree. Rhizosphere soil was collected using the root-shaking method. All specimens were conveyed to the lab in portable coolers, and the root materials were used to determine arbuscular mycorrhizal fungi (AMF) colonization rates. After screening at 2 mm to eliminate pebbles and plant detritus, the soil specimens were partitioned into two groups. A subset was stored frozen at -20 °C for subsequent physicochemical and microbial analyses. The alternative subset was air-dried to evaluate AMF spore density and diverse soil physicochemical characteristics.

### Measurement of soil P fractionation

Rhizosphere soil P fractions were determined using a sequential biological-based method (DeLuca et al., 2015), which defines operationally extracted fractions and their plant acquisition pathways (**Table 1**). Briefly, 0.5 g of fresh soil was individually subjected to treatment with 10 mL of 0.01 mol·L^-1^ CaCl_2_ for CaCl_2_-P, 0.01 mol·L^-1^ citrate for Citrate-P, 0.02 EU·mL^-1^ enzyme cocktail for Enzyme-P, and 1 mol·L^-1^ HCl for HCl-P. After shaking at 180 rpm and 25 °C for 3 h, the mixtures were centrifuged at 10,000 rpm for 1 min. P in the collected supernatants was measured using the malachite green colorimetric technique at 630 nm.

**Table 1:** Correspondence between the operationally defined phosphorus (P) fractions extracted by the sequential biological-based method and their associated plant acquisition pathways.

Soil P fractions	Extracting agent	Ecological interpretation	Plant acquisition pathway
CaCl_2_-P	0.01 mol·L^-1^ mM calcium chloride	Soluble, readily available P in soil solution	Direct uptake via fine root
Citrate-P	0.01 mol·L^-1^citric acid	P bound to Fe/Al oxides or chelated by organic anions	Roots solubilize insoluble inorganic phosphorus by secreting organic acids for uptake
Enzyme-P	phosphatase and phytase (0.02 enzyme units ml^-1^)	Labile organic P compounds hydrolyzable by root extracellular enzymes	Roots mineralize organic phosphorus through phosphatase secretion for uptake
HCl-P	1 mol·L^-1^ HCl	Predominantly apatite-bound or other acid-soluble mineral P	Roots mobilize sparingly soluble inorganic phosphorus through proton secretion for uptake

### Soil physicochemical properties

SWC was assessed using the oven-drying technique. pH was assessed utilizing a pH meter with a soil-to-water ratio of 1:2.5 (w/v) (Peng and Wang, 2016). The soil total N (TN) was measured using a modified Kjeldahl method (Li et al., 2024). Total P (TP) was analyzed by molybdenum blue colorimetry following H_2_SO_4_-H_2_O_2_ digestion. Soil dissolved organic carbon (DOC) and organic nitrogen (DON) were quantified using a total organic carbon analyzer (Shimadzu TOC-V CPH, Japan). Soil ammonium nitrogen (NH_4_^+^-N) and nitrate nitrogen (NO_3_^–^-N) were quantified via KCl extraction (soil-to-solution ratio = 1:10), they were determined by the azo-cyanine blue colorimetric method and ultraviolet spectrophotometry, respectively (Lu, 2000).

### Soil microbial traits

Microbial biomass C and N (MBC/MBN) were quantified through chloroform fumigation-extraction methodology (Brookes et al., 1985). MBC and MBN were quantified by a TOC analyzer. Soil acid phosphatase activity (ACP) was assayed by p-nitrophenyl phosphate colorimetry (Schneider et al., 2000). Arbuscular mycorrhizal fungi (AMF) colonization rates were determined by acid fuchsin staining and the grid-line intersect method (Kormanik et al., 1980). AMF spore density (SD) was quantified using wet sieving and decanting followed by sucrose density gradient centrifugation (An et al., 1990).

### Data analysis

All data underwent homogeneity of variance testing; mathematical transformations were applied when necessary. One-way analyses of variance (ANOVA) with Tukey *post-hoc* tests assessed differences in the soil P fractions, soil physicochemical properties, microbial biomass, ACP, AMF colonization rate, and spore density among the forest stands. Two-way ANOVA evaluated the effects of forest stands and season on soil P fractions, physicochemical properties, and microbial traits. Pearson correlation analysis assessed the relationships among soil P fractions, physicochemical features, and microbiological traits. Additionally, random forest and linear mixed-effects models were utilized to assess soil and microbial drivers relative importance and contributions to different P fractions using the “rfPermute” package (version 2.5.5) and “lmerTest” package, respectively. PLS-PM assessed the direct and indirect impacts of biotic and abiotic factors on P fractions across forest types by using the ‘plspm’ package (version 4.5.1).

## Results

### Rhizosphere soil properties

**Figure 2:**
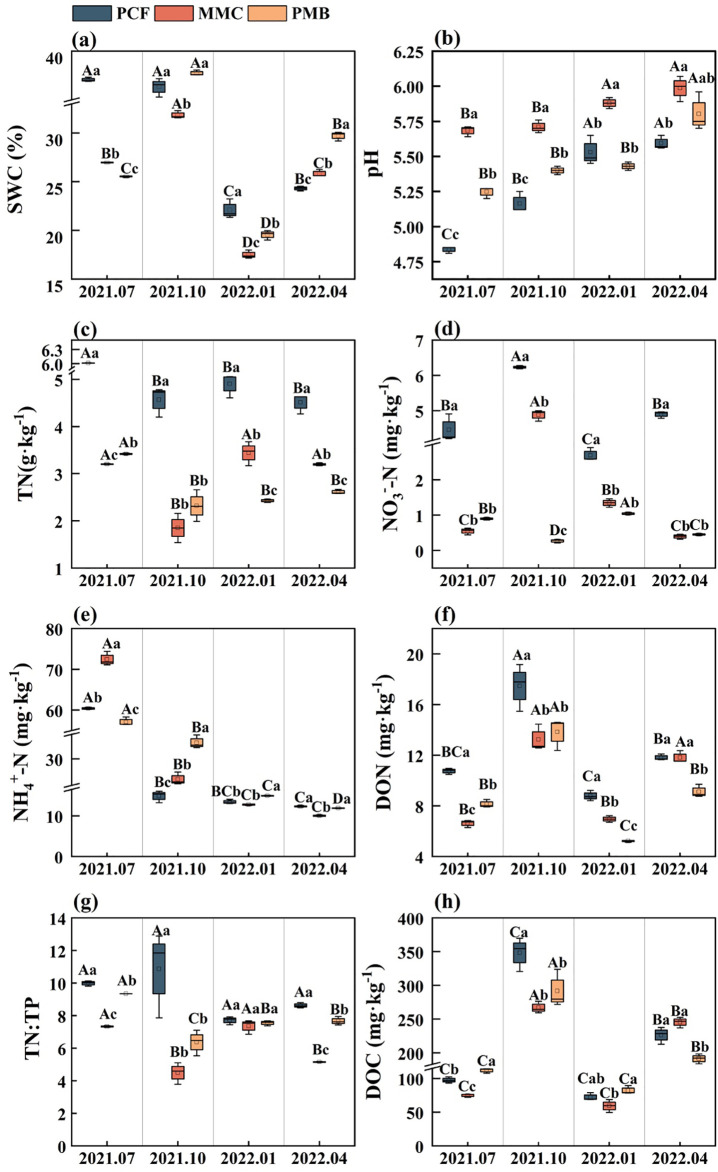
Influence of Moso bamboo expansion on rhizospheric characteristics of Chinese fir forests across seasons. **(a)** SWC; **(b)** pH; **(c)** TN; **(d)** NO3--N; **(e)** NH_4_^+^-N; **(f)** DON; **(g)** TN:TP; **(h)** DOC. Uppercase letters denote significant seasonal variations within a forest stand, whereas lowercase letters represent differences between forest stands within a given season. SWC, soil water content; TN, soil total nitrogen; NO_3_^-^-N, nitrate nitrogen; NH_4_^+^-N, ammoniacal nitrogen; DON, dissolved organic nitrogen; TN:TP, the ratio of total nitrogen to total phosphorus; DOC, dissolved organic carbon.

Compared with PCF, MMC and PMB decreased TN (29.0%–59.5%) and NO_3_^–^-N (21.7%–95.7%) in all four seasons (*p<* 0.05, **Figure 2**). MMC and PMB significantly reduced SWC by 11.5%–31.1% in summer and winter; DON in summer, autumn, and winter; NH_4_^+^-N content in spring; and significantly increased soil pH in spring, summer, and autumn (*p* < 0.05, **Figure 2**). In spring, soil pH was significantly higher in all three forest stands than in summer and autumn (*p* < 0.05, **Figure 2**). Conversely, SWC, DOC, and DON were highest in autumn (*p* < 0.05, **Figure 2**). Additionally, TN and ammonium N content were significantly higher in summer than in other seasons, specifically in pure Chinese fir and Moso bamboo forests (*p* < 0.05, **Figure 2**). Repeated-measures two-way ANOVA revealed that bamboo expansion, season, and their interaction profoundly influenced soil pH, SWC, TN, TN: TP, NH_4_^+^-N, NO_3_^–^-N, DON, and DOC (*p* < 0.05, **Table 2**).

**Table 2:** A repeated-measures two-way ANOVA was performed to assess the effects of bamboo expansion, season, and their interaction on rhizosphere soil physicochemical properties, microbial characteristics, and P fractions.

Source of variations	Moso bamboo expansion		Seasons		Interaction	
	F	*P*	F	*P*	F	*P*
pH	189.88	<0.001	105.53	<0.001	11.99	<0.001
SWC	230.86	<0.001	1492.32	<0.001	165.50	<0.001
TN	471.47	<0.001	62.69	<0.001	14.74	<0.001
TN: TP	43.794	<0.001	8.276	<0.001	7.87	<0.001
NH_4_^+^-N	82.74	<0.001	5222.51	<0.001	132.04	<0.001
NO_3_^–^-N	2240.72	<0.001	395.81	<0.001	265.39	<0.001
DON	55.28	<0.001	175.86	<0.001	6.88	<0.001
DOC	11.42	<0.001	645.72	<0.001	14.25	<0.001
MBC	0.42	0.665	94.72	<0.001	5.79	0.001
MBN	3.46	0.048	22.26	<0.001	11.94	<0.001
MBC: MBN	0.18	0.836	17.003	<0.001	4.475	0.004
ACP	0.67	0.521	2.65	0.072	1.79	0.145
SD	94.65	<0.001	54.22	<0.001	19.26	<0.001
AMF colonization rate	141.35	<0.001	101.61	<0.001	8.49	<0.001
TP	178.77	<0.001	21.22	<0.001	27.35	<0.001
CaCl_2_-P	59.47	<0.001	39.64	<0.001	25.06	<0.001
Citrate-P	76.04	<0.001	26.73	<0.001	6.79	<0.001
Enzyme-P	150.374	<0.001	77.88	<0.001	37.03	<0.001
HCl-P	105.894	<0.001	25.30	<0.001	37.82	<0.001
CaCl_2_-P:TP	15.152	<0.001	32.953	<0.001	26.118	<0.001
Citrate-P:TP	16.289	<0.001	24.8	<0.001	5	0.002
Enzyme-P:TP	46.991	<0.001	46.894	<0.001	21.117	<0.001
HCl-P:TP	12.456	<0.001	23.818	<0.001	22.167	<0.001

SWC, soil water content; TN, soil total nitrogen; NH_4_^+^-N, ammoniacal nitrogen; NO_3_^-^-N, nitrate nitrogen; DON, dissolved organic nitrogen; DOC, dissolved organic carbon; MBC, microbial biomass carbon; MBN, microbial biomass nitrogen; ACP, acid phosphatase activity; SD, AMF spore density; TP, soil total phosphorus.

### Rhizosphere microbial traits

**Figure 3:**
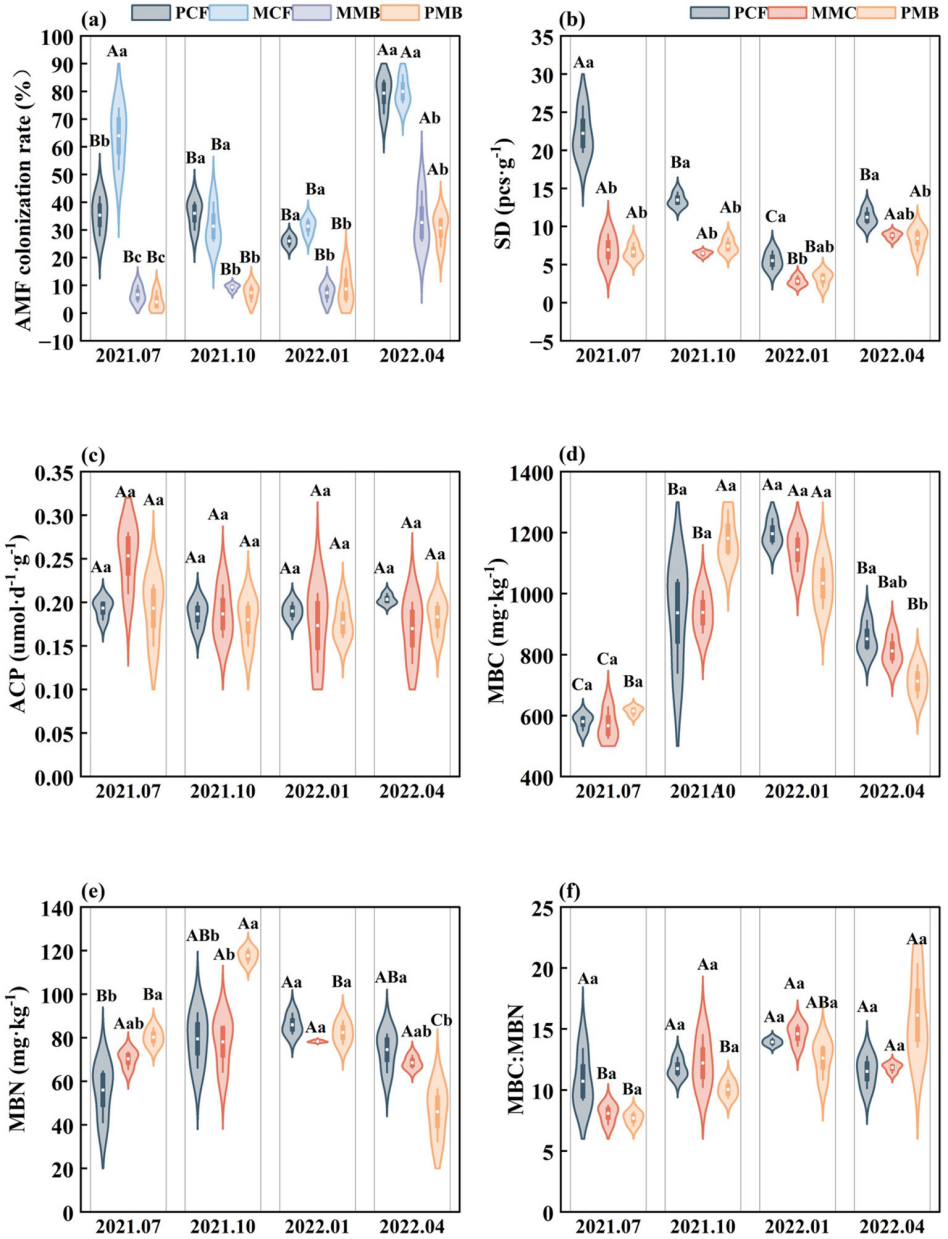
Influence of Moso bamboo expansion on rhizospheric microbial characteristics of Chinese fir forests across seasons. **(a)** AMF colonization rate; **(b)** SD; **(c)** ACP; **(d)** MBC; **(e)** MBN; **(f)** MBC:MBN. Capital letters denote seasonal marked variations within a forest stand; lowercase letters signify notable variances between forest stands within a season. SD, AMF spore density; ACP, acid phosphatase activity; MBC, microbial biomass carbon; MBN, microbial biomass nitrogen.

The rhizosphere soil of both the mixed forests and pure Moso bamboo forests exhibited significantly low AMF spore density (45.1%–70.1%) during the summer and autumn seasons compared to pure Chinese fir forests (*p<* 0.05, **Figure 3**). However, the colonization rate of AMF in the Moso bamboo plots decreased compared to the Chinese fir forests (*p<* 0.05, **Figure 3**). The MBC in pure Moso bamboo forests was significantly lower than that in pure Chinese fir forests during spring, while no significant change was observed in mixed forests. The MBN in pure Moso bamboo forests was significantly higher than that in pure Chinese fir forests during summer and autumn but showed a significant decrease in spring (*p<* 0.05, **Figure 3**). There were no significant differences in ACP activity among the other tree species (*p* > 0.05, **Figure 3**). AMF spore density peaked in summer across all stands. In contrast, MBC and MBN were highest in autumn (**Figure 3**). The repeated-measures two-way ANOVA revealed that bamboo expansion, season, and their interaction significantly affected MBN and AMF spore density and colonization rate (*p* < 0.05, **Table 2**).

### Rhizosphere P fractions

**Figure 4:**
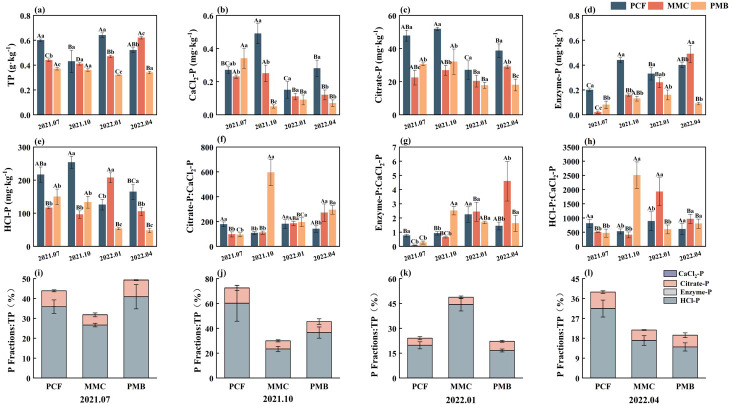
Influence of Moso bamboo development into Chinese fir forests on **(a)** TP; individual phosphorus fractions **(b–e)**, ratios of other P fractions to CaCl_2_-P **(f–h)**, and the proportional contributions of these fractions to total phosphorus **(i–l)**. Significant variations between forest stands within a season are indicated by lowercase letters, while significant differences between seasons within a forest stand are indicated by uppercase letters. TP, soil total phosphorus; MCF, Mixed Chinese fir forest; MMB, Mixed Moso bamboo forest.

Compared to the PCF, both MMC and PMB significantly reduced soil TP content in summer and winter, with the lowest values observed in PMB (*p* < 0.05; **Figure 4**). Across all three forest stands, HCl-P constituted the largest proportion of bioavailable P, followed by Citrate-P, while CaCl_2_-P and Enzyme-P accounted for relatively minor proportions—a pattern that remained consistent across seasons (**Figure 4**).

During spring and autumn, MMC significantly decreased CaCl_2_-P by 13.6%–49.3%, while Citrate-P (24.9%–53.1%), HCl-P (36% - 62.1%) contents were significantly decreased in summer, autumn, and spring relative to the PCF. PMB significantly reduced Enzyme-P (51.5%–77.5%) and HCl-P (30.8%–70.9%) across all four seasons, significantly decreased Citrate-P (36.1% - 53.5%) during spring, summer, and autumn, while only significantly lowered CaCl_2_-P in spring (75.0%) and autumn (89.8%). (*p* < 0.05; **Figure 4**). Compared with PCF, PMB significantly reduced the ratios of Citrate−P to CaCl_2_−P, Enzyme−P to CaCl_2_−P, and HCl−P to CaCl_2_−P in summer, whereas these ratios were significantly increased in autumn and spring (*p* < 0.05; **Figure 4**).

CaCl_2_-P, Citrate-P, and HCl-P were markedly elevated during summer–autumn than winter–spring across the PCF and PMF stands, while Enzyme-P showed an inverse seasonal pattern with peak values in winter–spring across the PCF and PMB stands (*p* < 0.05; **Figure 4**). In terms of seasonal dynamics, CaCl_2_-P, Citrate-P, and HCl-P were markedly elevated during summer–autumn compared to winter–spring across both the PCF and PMB stands. In contrast, Enzyme-P exhibited an inverse pattern, with peak values occurring in winter–spring across the PCF and PMB stands (*p* < 0.05; **Figure 4**). The repeated-measures two-way ANOVA revealed that bamboo expansion, season, and their interaction significantly influenced all P fractions (*p* < 0.05, **Table 2**).

### Drivers of soil P fraction dynamics

**Figure 5:**
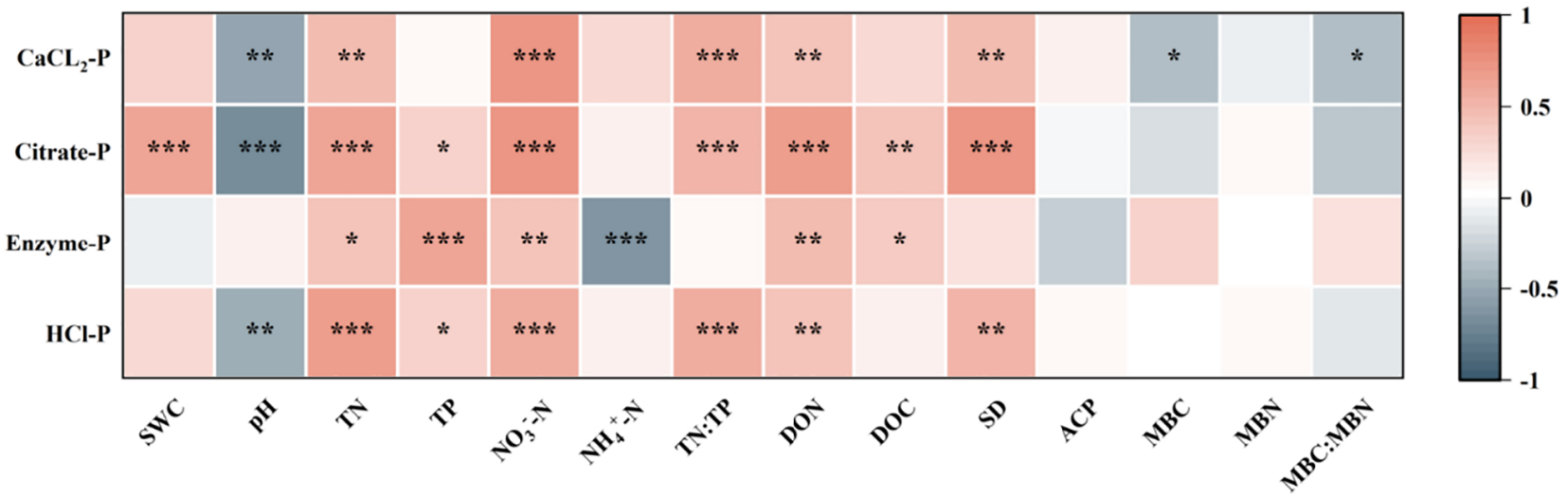
Correlations between rhizosphere soil phosphorus fractions, soil physicochemical properties, and microbial traits across different seasons and forest stands. *p < 0.005, **p < 0.001; ***p < 0.0001. SWC, soil water content; TN, soil total nitrogen; TP, soil total phosphorus; NO_3_^-^-N, nitrate nitrogen; NH_4_^+^-N, ammoniacal nitrogen; TN:TP, the ratio of total nitrogen to total phosphorus; DON, dissolved organic nitrogen; DOC, dissolved organic carbon; SD, AMF spore density; ACP, acid phosphatase activity; MBC, microbial biomass carbon; MBN, microbial biomass nitrogen.

All soil P fractions exhibited substantial positive associations with TN, NO_3_^–^-N, and DON (*p* < 0.05; **Figure 5**). Moreover, CaCl_2_-P, Citrate-P, and HCl-P exhibited an advantageous connection with AMF spore density while demonstrating a negative correlation with soil pH (*p* < 0.05; **Figure 5**).

**Figure 6:**
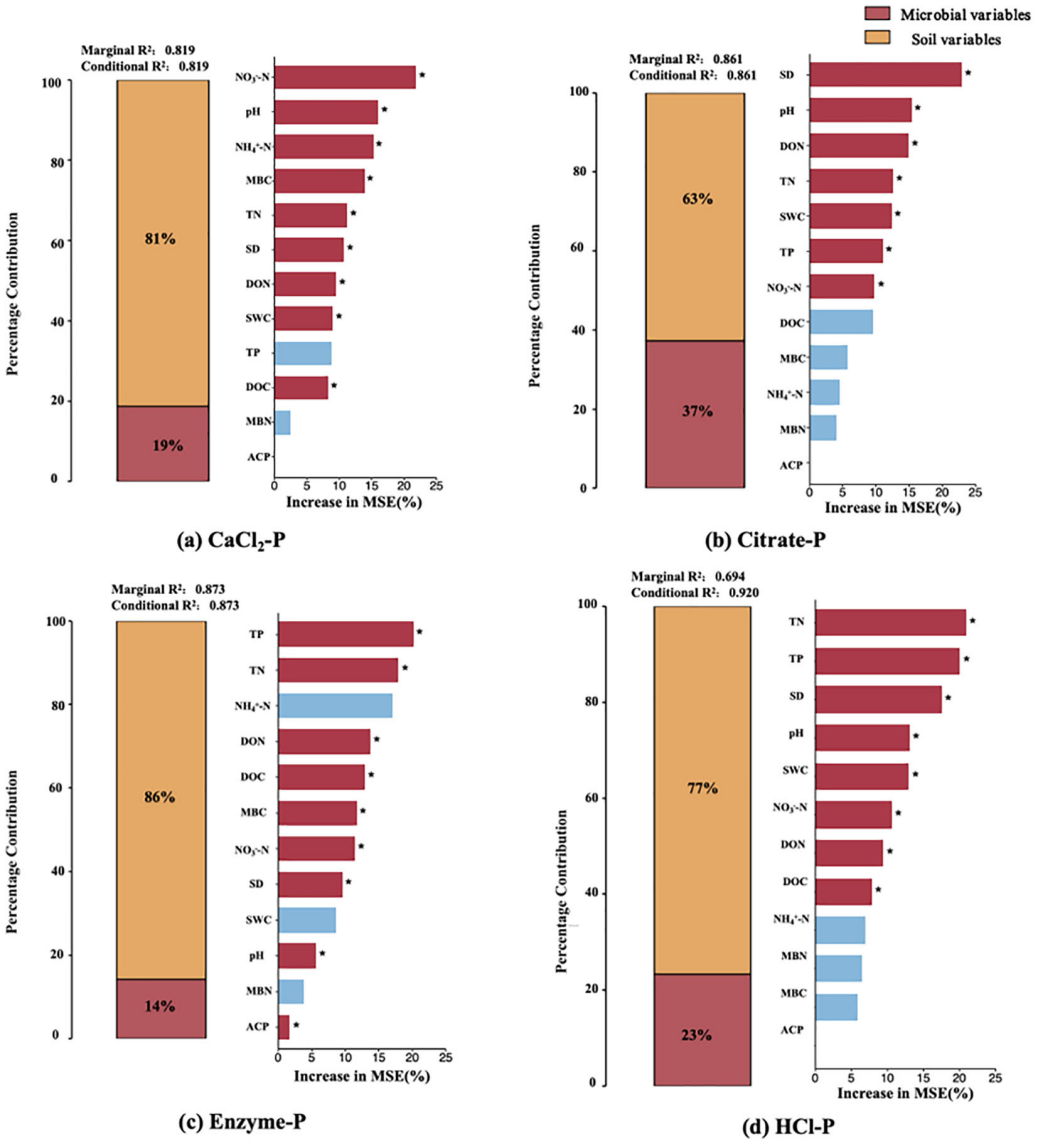
Analyses applying random forest and mixed-effects models to assess soil physicochemical and microbial influences on rhizosphere phosphorus fractions: **(a)** CaCl2-P, **(b)** Citrate-P, **(c)** Enzyme-P, and **(d)** HCl-P. *p < 0.005. TP, soil total phosphorus; TN, soil total nitrogen; NH_4_^+^-N, ammoniacal nitrogen; DON, dissolved organic nitrogen; DOC, dissolved organic carbon; MBC, microbial biomass carbon; NO_3_^-^-N, nitrate nitrogen; SD, AMF spore density; SWC, soil water content; MBN, microbial biomass nitrogen; ACP, acid phosphatase activity.

**Figure 7:**
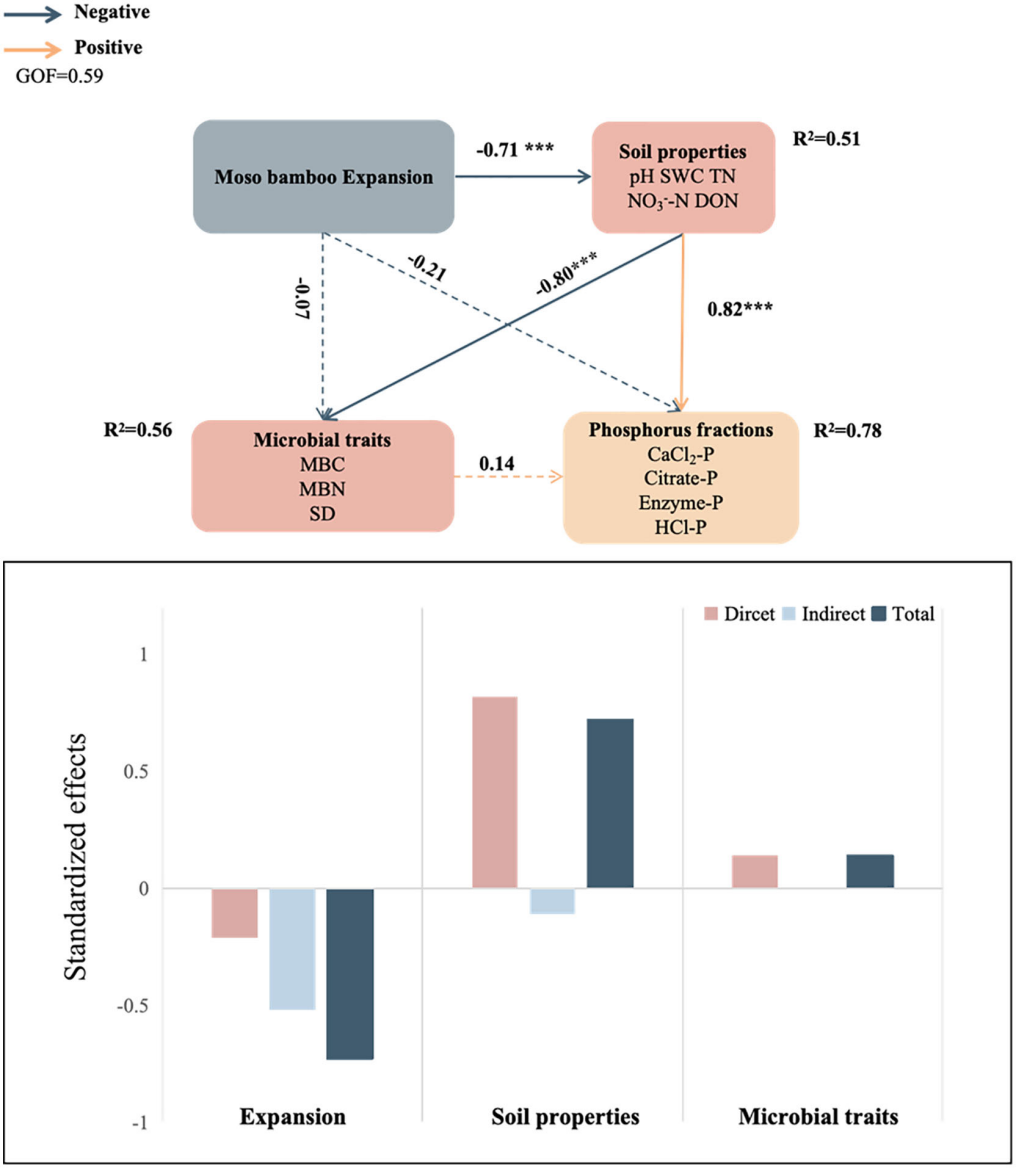
PLS-PM analysis revealing the direct and indirect impacts of biotic and abiotic factors on rhizosphere soil phosphorus fractions during Moso bamboo forests’ expansion into Chinese fir forests. In this diagram, red and blue lines denote positive and negative correlations, respectively, while solid and dashed lines indicate significant and non-significant pathways. Adjacent to each arrow, numerical values display standardized path coefficients, and the arrows’ thickness corresponds to the association’s magnitude *p < 0.05, **p < 0.01, ***p < 0.001. SWC, soil water content; TN, soil total nitrogen; NO_3_^-^-N, nitrate nitrogen; DON, dissolved organic nitrogen.

Hierarchical partitioning within the mixed-effects model revealed that soil physicochemical properties accounted for substantially greater variance (62.7%–85.9%) in all four P fractions compared to microbial factors (14.1%–37.3%), highlighting the predominant role of abiotic drivers over biological processes in regulating P speciation (**Figure 6**). The random forest analysis further identified NO_3_^–^-N and pH as the most important factors influencing CaCl_2_-P, while AMF spore density and pH were the dominant predictors for Citrate-P. TP and TN exerted the strongest effects on Enzyme-P. In contrast, TN, TP, AMF spore density, and pH were the primary factors governing HCl-P dynamics (**Figure 6**). PLS-PM indicated that both abiotic (pH, SWC, and N forms) and biotic factors (microbial biomass andAMF spore density) acted synergistically to regulate soil P fractions.Among these, soil N components (TN, NO_3_^–^-N, TN: TP, DON, and NH_4_^+^-N) served as the strongest positive direct drivers, while Moso bamboo expansion exhibited consistent negative direct effects (**Figure 7**).

## Discussion

### Reduction of rhizosphere P pools accompanying bamboo expansion

This study provides the first direct evidence that Moso bamboo expansion into Chinese fir forests markedly reduces total P and all measured bioavailable P fractions in rhizosphere soil, fully supporting the first hypothesis. Consistent with this, Wu et al. (2018) documented a reduction in total P in bulk soil following bamboo growth within coniferous forests. In contrast, expansion into broad-leaved forests generally does not affect total P levels and yields varying outcomes in bioavailable P (Yang et al., 2024). These discrepancies underscore the pivotal role of forest types and soil substrates role in governing P dynamics. Consequently, systematic comparisons across forest and soil types are urgently needed to clarify the generality of bamboo-driven P reduction.

The expansion of Moso bamboo forests caused a substantial decline in soil CaCl_2_-P content (**Figure 4**), presumably owing to heightened nutritional requirements from both bamboo and fir in mixed stands (Wu et al., 2019). Additionally, the elevated biomass of Moso bamboo compared to other subtropical tree types augments its absorption of labile P (Song et al., 2017; Xu et al., 2020), further reducing rhizosphere available P. However, in summer, the CaCl_2_-P content in the rhizosphere of pure Moso bamboo forests was significantly higher than that in pure Chinese fir forests and Moso bamboo–Chinese fir mixed forests. This may be attributed to higher rhizospheric microbial activity in pure bamboo stands during the summer, which enhances the mobilization of this P fraction. Therefore, we acknowledge that this conclusion is based on a limited set of measured microbial attributes (MBC, MBN, ACP, AMF). A more comprehensive profiling of the microbial community (e.g., via high-throughput sequencing) and its functional potential might reveal a greater contribution of microbial processes to P cycling than currently estimated. Therefore, the role of microbial factors in our system may be underrepresented.

The reduction in Citrate-P and HCl-P may be attributed to Moso bamboo’s extensive rhizome–root system; substantial belowground biomass improves P-acquisition efficiency through fine roots (Song et al., 2017, Song et al., 2020; Ye et al., 2021), thereby diminishing investment in exudate-based P-mobilizing strategies and resulting in the decline of these P fractions. The observed rise in soil pH following bamboo expansion supports this interpretation (**Figures 2**, **5**). This was because soil pH impacts the composition and variety of P-solubilizing microbes and affects iron and aluminum oxides, which in turn influence soil P fractions (Hou et al., 2018b; Wu et al., 2020; Wan et al., 2021; Zhou et al., 2020). However, some studies have found that Moso bamboo expansion can lead to a decrease in soil pH (Yang et al., 2024). The divergence in these findings may be attributed to variations in geographical environments or differences in the conditions of the invaded forest stands. The pH increase in our study may be related to specific rhizosphere processes of Moso bamboo, such as distinct exudate inputs, alterations in microbial community structure, or differences in organic acid metabolism pathways, the precise mechanisms of which require further investigation. Nevertheless, the observed pH increase was consistent with the trends in Citrate-P and HCl-P reported in our study, jointly supporting the core hypothesis that bamboo expansion influences phosphorus availability by modifying the rhizosphere microenvironment.

Decline in Enzyme-P may be attributed to the higher C:P ratio of Moso bamboo litter compared to Chinese fir litter (Song et al., 2015), which stimulates microbial P immobilization. Additionally, rich in recalcitrant lignin, bamboo litter hinders the breakdown of organic substances, reducing enzymatically accessible P renewal (Li et al., 2019; Liu et al., 2024). Diminished ACP activity and microbial populations across pure bamboo and mixed soils, versus pure Chinese fir, partially reinforce this process (**Figure 3**). In summary, the reduction in both total and bioavailable P during Moso bamboo expansion indicates a shift toward a “high turnover, low storage” P-cycling strategy, aligning with earlier observations (Li et al., 2021).

In all three forest stands, the high proportions of HCl-P and Citrate-P in TP, along with their ratios to CaCl_2_-P, exceeded 1 (**Figure 4**), suggesting that both Moso bamboo and Chinese fir primarily rely on root-exudate-mediated P activation for P acquisition—a conclusion aligned with Yang et al. (2024). Furthermore, the significantly lower AMF colonization rate in Moso bamboo than in Chinese fir indicates that the latter relies more heavily on the mycorrhizal pathway for P acquisition. However, as these results only indirectly reflect plant P uptake pathways, more direct methodologies are needed to clarify P acquisition strategies and better understand the P-cycling dynamics during bamboo forest expansion. Furthermore, if some mixed stands were not formed by bamboo invasion, the conclusion that “bamboo expansion leads to a decrease in rhizosphere phosphorus content” requires further verification, and the underlying mechanisms warrant further investigation.

### Moso bamboo expansion drives persistent rhizospheric P decline despite seasonal dynamics

CaCl_2_-P, Citrate-P, and HCl-P levels were notably elevated in summer and fall compared to winter and spring across Moso bamboo, Chinese fir, and mixed forest stands (**Figure 4**), corroborating the second hypothesis. This seasonal pattern is driven by two interconnected mechanisms: (1) Optimal summer–autumn climatic conditions and elevated microbial activity, accelerating organic matter decomposition and subsequent release of labile P (e.g., CaCl_2_-P). In this study, the higher MBC content in autumn, which showed a significantly positive correlation with CaCl_2_–P (**Figures 3**, **4**), supports this mechanism. (2) During the growing season (summer–autumn), root organic acid exudation rates are higher than they are in winter–spring, and high humidity reduced soil pH, thereby increasing the abundance of Fe(III)-reducing bacteria (e.g., *Geobacter*) (Hou et al., 2018a); this enhances the dissolution of Citrate-P and HCl-P. Lower pH observed in summer and autumn compared to winter and spring further supports this mechanism (**Figure 2**).

In all three forest types, the rhizosphere Enzyme-P concentration was markedly decreased in summer compared to other seasons (**Figure 4**). This pattern can be attributed to two key mechanisms: (1) concentrated litterfall in autumn and winter increases organic P input, whereas summer—being the peak growing season—exhibits reduced organic P input, leading to lower Enzyme-P; and (2) lower temperatures suppress acid phosphatase activity, thereby limiting the mineralization of Enzyme-P. This study also found higher ACP activity in summer compared to other seasons (**Figure 3**), further supporting the second mechanism. Despite distinct seasonal patterns in rhizosphere P fractions across the forest types, the overall trend of expansion-induced P reduction from bamboo to fir stands remained unaltered.

However, we acknowledge that the seasonal effects discussed here also incorporate between-year variability and potential influences from management activities (e.g., bamboo shoot harvesting typically occurring in winter–spring) (Liu et al., 2023), which may confound the interpretation of purely seasonal drivers.

## Conclusion

**Figure 8:**
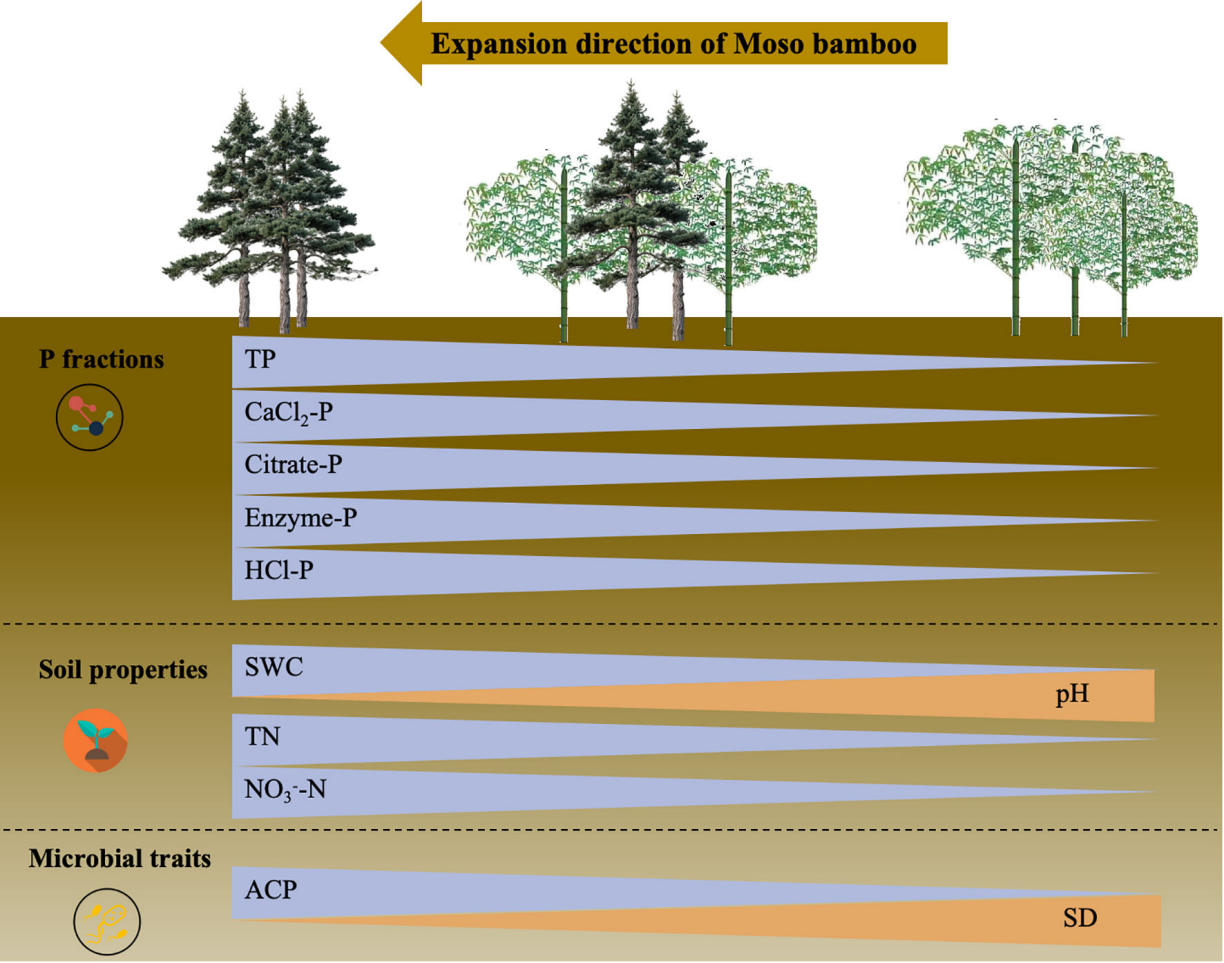
A conceptual graphic depicting the alterations in soil phosphorus fractions and their principal driving forces after the expansion of Moso bamboo into Chinese fir woods. TP, soil total phosphorus; SWC, soil water content; TN, soil total nitrogen; NO_3_^-^-N, nitrate nitrogen; ACP, acid phosphatase activity; SD, AMF spore density.

In this study, a space-for-time replacement method was used to demonstrate, for the first time, the dynamic evolution of rhizosphere P pools throughout the spread of Moso bamboo inside Chinese fir woodlands (**Figure 8**). Total P and bioavailable fractions demonstrated a significant decrease along the expansion gradient. This pattern remained stable across seasons. Reduction was directly linked to bamboo’s high root biomass and fine-root foraging strategy for efficient P acquisition. Rhizosphere Citrate-P and HCl-P peaked in summer–autumn while Enzyme-P peaked in winter–spring, demonstrating synchronous P-fraction seasonality across forest types. These findings advance niche competition theory by elucidating P-form turnover during bamboo expansion and guide seasonal P management for subtropical plantations, supporting sustainable forestry under “dual carbon” goals. To resolve critical knowledge gaps, subsequent research must dissect interspecific variation in root-foraging precision and leaf-P resorption efficiency between Moso bamboo and Chinese fir, thereby refining P cycling models for subtropical forests.

## Data Availability

The original contributions presented in the study are included in the article/supplementary material. Further inquiries can be directed to the corresponding author.
